# Extra-uterine placental transfusion and intact-cord stabilisation of infants in caesarean sections: an intervention development and pilot-study (INTACT-1)

**DOI:** 10.1186/s12884-025-07641-w

**Published:** 2025-05-09

**Authors:** Elisabeth Saether, Ola Andersson, Solveig Bjellmo, Stine Bernitz, Friedrich Reinhart-Van Gülpen, Tor Åge Myklebust, Solhild Stridsklev, Beate Horsberg Eriksen

**Affiliations:** 1 Department of Obstetrics and Gynaecology, Clinic Ålesund, Møre and Romsdal Hospital Trust, Aalesund, Norway; 2 Faculty of Medicine and Health Sciences, Department of Clinical and Molecular Medicine, Trondheim, Norway , Norwegian University of Science and Technology, Trondheim, Norway; 3 Department of Neonatology, Skåne University Hospital, Malmö/Lund, Sweden; 4 Department of Clinical Sciences Lund, Pediatrics/Neonatology, Lund University, Lund, Sweden; 5 Faculty of Medicine and Health Sciences, Faculty Administration, Norwegian University of Science and Technology, Trondheim, Norway; 6 Department of Obstetrics and Gynaecology, Østfold Hospital Trust, Grålum, Norway; 7 Faculty of Health Sciences, Department of Health Promotion, Oslo Metropolitan University, Oslo, Norway; 8 Department of Paediatrics, Møre and Romsdal Hospital Trust, Ålesund, Norway; 9 Department of Registration, Cancer Registry Norway, Norwegian Institute of Public Health, Oslo, Norway; 10 Department of Research and Innovation, Møre and Romsdal Hospital Trust, Ålesund, Norway; 11 Department of Obstetrics and Gynaecology, St.Olavs Hospital, Trondheim, Norway; 12 Faculty of Medicine and Health Sciences, Department of Public Health and Nursing, Norwegian University of Science and Technology, Trondheim, Norway

**Keywords:** Umbilical cord clamping, Intact cord stabilisation, Placental transfusion, Infant, Caesarean section

## Abstract

**Background:**

Keeping the umbilical cord intact the first minutes after delivery is beneficial for both term and preterm infants. However, this may be challenging in caesarean sections (CS) due to lack of mobile resuscitation equipment, maintenance of sterility or concern for excessive maternal blood loss. The objective of this study was to develop and pilot-test extra-uterine placental transfusion and intact-cord stabilisation of infants in CS.

**Methods:**

The intervention development process (phase 1) covered: (A) placenta delivery without cord clamping, (B) intact-cord stabilisation of the infant and (C) physiology-based cord clamping. Different scenarios were tested through in-situ simulation and adjusted through multiple feedback rounds. The involved staff were trained prior to pilot-testing (phase 2). Women having a CS in regional anaesthesia, expecting a term or near-term singleton infant were included in the pilot-study after written consent. Primary outcome was the proportion of successfully completed interventions. For assessment of safety, maternal estimated intraoperative blood loss, infant 5-minute Apgar scores and infant rectal temperature during stabilisation were compared to pre-defined accept criteria. Dry-electrode ECG (NeoBeat™) was used for early detection of infant heart rate. Any respiratory support was registered. Early skin-to-skin contact between mother and infant was attempted for vigorous infants.

**Results:**

A detailed intervention protocol was developed and tested. Twenty-nine mother-infant-dyads were included in the pilot study. Gestational age ranged from 37 to 42 weeks. The intervention was successfully completed in 26 of 29 cases, of which 31% were planned CS. Median (SD) infant heart rates at one and five minutes were 159 (32) and 168 (21) beats per minute respectively. Eight infants (28%) had intact-cord respiratory support. One infant had a 5-minute Apgar score < 7 and three infants (10%) had rectal temperatures below 36.5 °C during the first 10–15 min after birth. Three mothers (10%) had estimated intraoperative blood loss > 1000 ml.

**Conclusion:**

Extra-uterine placental transfusion to facilitate intact-cord stabilisation and physiology-based cord clamping for term and near-term infants delivered by CS was feasible according to predefined accept criteria. Further investigation of safety of this complex intervention in larger, comparative studies is warranted.

**Trial registration:**

Regional Committee for Medical Research Ethics Central Norway (REK-Midt), #399101.

**Supplementary Information:**

The online version contains supplementary material available at 10.1186/s12884-025-07641-w.

## Background

Although cutting the umbilical cord at birth is the most common intervention in obstetrics, timing is still under debate. Keeping the umbilical cord intact the first minutes after birth is beneficial for both term and preterm infants [1], however still not routinely offered to all infants [2, 3, 4]. In emergency or preterm caesarean sections (CS), clinicians may have to choose between delaying cord clamping and delaying stabilisation of the infant, since equipment for stabilisation often is placed at a distance from the operating table (2–4). Both the World Health Organization (WHO) and the European Resuscitation Council (ERC) recommend delayed cord clamping (DCC; 1–3 min after birth) for all births during stabilisation [5, 6]. Early cord clamping (ECC; less than one minute after birth) may result in a 25–33% reduction in total circulating blood volume and up to 33% reduction in red blood cells [7, 8, 9] and is not recommended unless the infant needs to be moved for resuscitation [5, 6]. In preterm infants, DCC may reduce intraventricular haemorrhage and need for early blood transfusions [10], increase stem cell transfer [11] and increase chances of survival [12, 13, 14]. In term infants, DCC ≥ 3 min reduces risk of iron deficiency the first 4–6 months [15, 16], anaemia at 8 and 12 months [17], and improves fine motor skills and social domain scores at 4 years of age [18]. DCC ≥ 5 min results in increased myelin in the brain at 4 and 12 months after birth compared to ECC [19, 20]. However, the optimal time to clamp the cord is still questioned [1, 21, 22].

Maintenance of cardiovascular stability during transition from intra- to extra-uterine life has gained more attention during the last decade [23, 24, 25]. ECC cuts off the major source of left ventricular preload to the heart, and this will in turn reduce cardiac output [24]. Experimental evidence suggests DCC until sufficient respiration and thereby pulmonary blood flow is established, securing preload from the perfused lungs to the left ventricle [26]. This is supported by studies on intact-cord stabilisation (ICS) of term and preterm infants, indicating that cord clamping should be physiology-based (after onset of respiration) rather than based on timing by a stopwatch [27, 28]. Until recently, placenta-to infant transfusion was thought to be primarily time- and gravity-dependent, but also varying with gestational age (GA) and mode of delivery [7, 29, 30, 31]. Blood flow in the umbilical vein and arteries after birth seems to be regulated by physiological factors, especially respiratory [24, 25], and is not necessarily related to cease of cord pulsations [23]. Some concern exists that lack of uterine contractions in CS may result in an infant-to-placenta transfusion if the cord is left unclamped [7, 32], but studies have demonstrated that placenta-to-infant transfusion happens during CS with DCC [33, 34, 35].

Infant heart rate (HR) during the first minutes after birth is the most sensitive clinical indicator to assess the infant’s condition and evaluate effect of treatment [21]. In Norway, the neonatal team is usually not present at the operating table when a CS is performed, thus transitional care is initiated by the attending midwife. Assessment of HR is commonly done by palpation of the umbilical cord, although this method is often inaccurate [36]. Both the ERC and The International Liaison Committee on Resuscitation (ILCOR) suggest using electrocardiography (ECG) for reliable estimation of HR during resuscitation [6, 37]. Wireless dry-electrode ECG has been tested for accuracy and sensitivity [38] and provide reliable HR readings earlier (5–10 s) than conventional ECG [39]. The device is easy to attach, but cannot be sterilised.

The current CS-rate in Norway is 16%, of which 65–70% are emergency CS [40]. An informal e-mail survey sent to Norwegian delivery units in March, 2021 revealed that ICS using mobile resuscitation equipment is not common in the operating room (OR). Several solutions have been tested for use in CS, ranging from different bedding combined with resuscitation equipment mounted on a portable pole to purpose-built mobile trolleys [41, 42]. The key to success is bringing the neonatal team into the OR for provision of immediate, transitional care [41, 42]. Known challenges are maintaining workflow and sterility in the surgical field and preventing infant hypothermia, which require advanced inter-professional collaboration, joint development of operating procedures and simulation-based training [43, 44]. The risk of bleeding from incision site while allowing for placental transfusion must be weighed against the benefits of DCC for the infant [43]. However, studies have found no difference in severe maternal haemorrhage (> 1000 ml) with DCC or physiology-based cord clamping (PBCC) during CS [34, 45, 46, 47].

Delivering the placenta before cord clamping was suggested as an alternative (extra-uterine) way of facilitating ICS during CS many years ago [48], and was recently tested for feasibility in two smaller studies [49, 50]. Obstetricians at our clinic had adopted this practice in cases of urgency for mother or infant (or both). However, no formal, preceding feasibility assessment of the intervention had been done. Hence, the purpose of this study was to develop and pilot-test extra-uterine placental transfusion combined with ICS and PBCC in term and near-term CS.

## Methods

We defined the combination of extra-uterine placental transfusion, intact-cord stabilisation and physiology-based cord clamping in CS as a complex intervention. The British Medical Research Council (MRC) has developed a framework for developing and testing complex health care interventions, consisting of four phases: (1) Development, (2) feasibility and piloting, (3) evaluation and (4) implementation testing [51]. Reporting was done according to guideline for intervention development studies in health research (GUIDED) and template for intervention description and replication (TIDieR) [52, 53]. For more details, refer Additional files 6 and 7.

### Phase 1 Intervention development

Intervention development studies may be defined as studies that “describe the rationale, decision making processes, methods and findings which occur between the idea or inception of an intervention until it is ready for formal feasibility, pilot or efficacy testing prior to a full trial or evaluation” [54], p.1.

### Identifying the evidence base

#### Extra-uterine placental transfusion and intact-cord stabilisation of the infant

We conducted a systematic literature review regarding whether extra-uterine placental transfusion may be a safe and feasible alternative to delayed cord clamping in CS. We searched the databases PubMed, EMBASE and Scopus, using the concepts “extra-uterine placental transfusion AND caesarean section”. A PRISMA flow chart is available as additional material (Additional file 1). Inclusion criteria were: (1) primary research, (2) human research, (3) describing extra-uterine placental transfusion, (4) and its impact on infant and/or mother. A total of four primary studies remained for critical appraisal, using a modified version of the CASP (Critical Appraisal Skills Programme) checklist for cohort studies [55]. The results are shown in Table 1.

**Table 1:** Critical appraisal of included studies

Author	Study design	Sample	Interventions	Outcome measures	Major findings	Critical appraisal
Landau et al. (54), 1950, United States	Observational quality improvement study	87 infants delivered by CS after introducing a new procedure.	Extra-uterine placental transfusion (placenta suspended above the infant). DCC when cord collapsed (*n* = 87). No control-group.	Placenta weight before and after suspension. Neonatal deaths caused by “secondary shock” (assumed to be Respiratory Distress Syndrome / haematogenic shock)	No incidences of “secondary shock” after implementing the procedure. Mortality rate was 2.3%. Reported overall mortality rate after CS at the time was 8–9%	Detailed description of the procedure. Maternal outcomes not reported. No mention of recruitment, inclusion criteria or confounders. No statistical analyses performed. Small sample size Do not qualify as evidence base for intervention development
Secher & Karlberg (55), 1962, Sweden / Denmark	Observational study	30 infants, 10 delivered by CS and 20 delivered vaginally (two control groups)	Extra-uterine placental transfusion after CS (*n* = 10) vs. after vaginal birth (*n* = 10). Cord temporarily clamped at birth, opened after suspending placenta above infant. Measured weight change of infant during transfusion. Control 2 (*n* = 10): Measured residual placenta blood volume after transfusion in vaginal birth.	Infant weight change from birth until cord clamping (during extra-uterine placental transfusion), estimated transfused blood volume	No “essential” difference in infant weight change between the groups. Correlation between birthweight and transfused blood volume.	Detailed description of the procedure. Infant and maternal outcomes not reported. No mention of recruitment, inclusion criteria or confounders. No statistical analyses performed. Small sample size Do not qualify as evidence base for intervention development
Kuehne et al. (47), 2018, Germany	Retrospective cohort study	60 preterm infants delivered by CS, gestational age less than 32 weeks (40 in the experimental group and 20 in the comparison group)	Extra-uterine placental transfusion and DCC after initiation of respiratory support, placenta kept above the infant (*n* = 40) vs. DCC/ umbilical cord milking before respiratory support (*n* = 20)	Apgar scores, cord-clamping time, grade of intraventricular haemorrhage, bronchopulmonary disease, necrotising enterocolitis or spontaneous intestinal perforation requiring surgery, peak serum-bilirubin levels, blood transfusions, survival until discharge. Subgroup: Infant saturation and HR during stabilisation	No statistically significant differences in outcome parameters between the groups.	Detailed description of the method used for the experimental group, not for the control group. Infant outcomes only. No adjustment for confounders. Non-randomised design, small sample size. Qualify as evidence base for intervention development
Welsh et al. (48), 2020 United States	Pilot study with matched historical control	30 mother-infant couples who underwent uncomplicated term CS in regional anaesthesia	DCC for 3–4 min after intact-cord placental delivery, placenta kept at same level as infant (*n* = 15) vs. ICC before 30 s (*n* = 15)	Safety: maternal estimated blood loss, newborn Apgar scores, temperatures, transcutaneous bilirubin levels, need for phototherapy, admission to NICU Feasibility: proportion of completed protocols, maternal satisfaction and clinician’s comfort with the procedure, mean placenta removal time, cord clamping time	No significant between-group differences in safety parameters. Protocol completed in 94% of deliveries. High maternal satisfaction. High clinician comfort.	Covers both maternal and infant outcomes. Detailed description of the method used. Small sample size. Non-randomised design, no mention or adjustment for confounders. Qualify as evidence base for intervention development

CS = Caesarean section, DCC = Delayed cord clamping, NICU = Neonatal Intensive Care Unit, ICC = Immediate cord clamping

Landau et al. [56] performed extra-uterine placental transfusion in a quality-improvement study including 87 infants delivered by CS. The objective was to reduce or prevent neonatal deaths. Secher and Karlberg [57] performed a pilot study, using a similar method. Twenty infants were weighed before and during extra-uterine placental transfusion. There were no reports on maternal outcome or infant follow up in these early studies to be used as evidence base for our intervention.

Kuehne et al. [49] compared extra-uterine placental transfusion to umbilical cord milking and DCC in a retrospective pilot study including 60 very preterm infants delivered by CS. There were no statistically significant differences in outcomes. Welsh et al. [50] performed an observational pilot study with a matched historical control, including 30 term infants delivered by elective CS. The objective was to determine whether extra-uterine placental transfusion was feasible and safe to support a larger study on the benefits of the method. There were no statistically significant differences between the groups. As the evidence base from this literature review was scarce and no firm conclusions could be drawn, it seemed justified to further investigate extra-uterine placental transfusion as an alternative to DCC in CS.

#### Stabilisation of newborn infants

The Norwegian Resuscitation Council updated their guidelines for neonatal stabilisation and resuscitation in 2021 [58], based on the ILCOR Summary Statement Paper [21] and the 2021 ERC Guideline [6]. Aspects especially relevant for our intervention development process were: (1) Assessment of the infant every 30–60 s, (2) infant heat preservation during stabilisation (36.5–37.5 °C), (3) respiratory support should be started within 60 s if necessary, and may be initiated with an intact umbilical cord, and (4) surveillance with ECG is recommended for HR-detection, dry-electrode ECG may be used immediately after birth.

#### Skin-to-skin contact between mother an infant in the operating room

Our hospital is Mother-Baby-Friendly-accredited, aiming at fulfilling the WHO 10 steps to successful breastfeeding. Step 4 recommends that health care professionals “*facilitate immediate and uninterrupted skin-to-skin contact (…) as soon as possible after birth”* [59]. During CS, non-separation and skin-to-skin contact (SSC) may be disturbed if the infant needs respiratory support, or the mother’s condition requires treatment. Prior to pilot testing the intervention, and for comparison, we investigated the degree of early SSC during CS at our hospital.

### Protocol development (the INTACT-intervention)

The intervention development was done at Møre and Romsdal Hospital Trust, Clinic Ålesund during spring 2022. In Norway, a neonatal team (paediatrician/paediatric registrar and specialist nurse) is only present during emergency or preterm CS, or when complications are anticipated in planned CS. All midwives and anaesthesiologists are trained in initial stabilisation and resuscitation of newborn infants, and a midwife is present at the operating table during all CS. The in-hospital neonatal team is available in few minutes if unforeseen complication should occur.

The intervention development team comprised experienced staff commonly involved in CS. The aim was to develop a protocol draft to be pilot-tested in a small cohort of mother-infant dyads (Phase 2). The protocol described by Welsh et al. [50] was chosen for further development, since their protocol resembled to some extent what was already done at our hospital, and it covered term infants.

Our protocol draft differed from the protocol by Welsh et al. on the following points: We decided to cover both planned and emergency CS (regional anaesthesia only). We would use dry-electrode ECG for early detection of infant HR in the surgical field. Instead of delivering the placenta as soon as possible, we would wait at least one minute after delivery to allow possible spontaneous separation. We incorporated sampling for cord blood gases from a pulsating umbilical cord (hospital routine). Instead of DCC for three minutes, we chose a physiology-based approach (after regular breathing and white/pulseless cord). We included intact-cord stabilisation for infants needing respiratory support, and early SSC for vigorous infants. A flowsheet visualising the intervention workflow was drafted for testing, accompanied with detailed role responsibilities. Seven workshops were held with participants from relevant clinical disciplines. The intervention components were tested and refined, and we identified barriers and enablers for implementation. The following challenges were identified for in-situ simulation testing:

- The mother’s thighs did not provide a firm surface for the infant, and this could possibly lead to obstruction of airways (infant falling between legs).
- Using non-sterile dry-electrode ECG (NeoBeat™) for infant surveillance would contaminate the midwife’s hands and potentially the sterile field/surgical team.
- Carrying both the infant and a tray containing a wet and slippery placenta could be challenging for the midwife.
- Presence of the paediatric registrar at the operating table would be valuable for initial assessment of the infant, but full scrubbing and gowning could be an obstacle if another emergency call needed attention.
- Prevention of infant hypothermia after CS was challenging already before study start and would need extra attention.
- There would be no extra personnel available for real-time data collection during CS. Tasks would have to be shared between the midwife, circulating OR-nurse and NICU-nurse.

### In-situ simulation

The simulation team comprised clinicians normally present at CS (midwife, paediatric registrar, obstetrician, obstetric registrar, anaesthesiologist, nurse anaesthetist, scrub nurse and OR circulating nurse). All sessions were done in-situ with CS set-up and equipment. We used a modified pit-stop model to visualise team set-up for each scenario (Additional files 2 and 3). The in-hospital simulation unit (VirtSim HMR) assisted in planning, supervision and evaluation. All sessions were moderated by certified facilitators and were videotaped using the SimCapture™ application (Laerdal Medical, Norway). A photographer was present for production of an instructional video.

Simulation also included testing of data collection tools and checklists to be used in the OR. After each scenario, the participants were invited to answer a simple two-item questionnaire regarding their experiences. Feedback was used to adjust subsequent scenarios, and was later systematised and incorporated in the protocol draft. Prior to pilot testing the draft was discussed with the research group and the user (patient-) representatives.

Eight in-situ simulation sessions were held, covering the following scenarios:

A. Planned term or near-term singleton CS.
B. Planned, near term twin CS.
C. Term, emergency CS grade 2 (within 30 min), anticipated vigorous infant.
D. Preterm, emergency CS grade 2, anticipated need for infant respiration support.
E. Early and uninterrupted SSC in the operating room (term, vigorous infant).

#### Feedback from simulation

Maintaining sterility:

- Covering all infants in a sterile plastic bag (SteriDrape™ Isolation bag 50 × 50 cm, 3 M, USA) could both prevent hypothermia and protect sterility if the midwife kept her hands inside the bag after attaching the NeoBeat™.
- Close collaboration between obstetrician, scrub nurse and midwife was needed for safe coverage of the infant, keeping the airways clear and avoiding contamination.
- An additional sterile bag was chosen for safe transfer of placenta, as it could easily be wrapped together with the infant in a sterile towel.
- The midwife should remove the outer pair of gloves before moving the infant/placenta.

Initial assessment, stabilisation and care of the infant.

- Two mattresses were tested for safe placement of the infant: A diaper changing mat 50 × 70 cm, and Vacuform™ 40 × 70 cm surgical cushion (B&W Schmidt, Germany). The latter mattress was chosen, as it proved easier to attach and interfered less with the mayo stands (instrument tables).
- If standing un-scrubbed at the mother’s head end, the paediatric registrar could assess the infant’s tone and colour and get early HR-readings from the NeoBeat™. Observations would support decisions on whether to abandon protocol.

Team collaboration and closed loop communication.

- Careful handling of syringes for blood sampling was required to avoid sharps injury.
- Facemasks and any extra noise in the operating room compromised real-time data collection, necessitating clear and focused closed loop communication.
- During planned CS, an extra person would be needed to assist the midwife in data collection at the resuscitation table.

Early and uninterrupted SSC between mother and infant:

- Placement of ECG-electrodes on the mother’s upper back/shoulders instead of on the chest provided more space for the infant. Prioritising one arm for IV (intravenous) lines and blood pressure cuff eased mother’s embracement of the infant.
- A convective temperature management system (Bair Hugger™, 3 M Health Care, US) and a modified three-layer non-woven leg warmer (Mölnlycke Barrier™, Germany) were tested for heat loss prevention. The latter was found easier to use (in combination with warm towels).
- For vigorous infants, a short welcoming cuddle could easily be incorporated to keep separation from the mother at a minimum.

Extra-uterine placental transfusion and twin CS:

- We found no safe and feasible way to handle intact-cord stabilisation of twins with a fused placenta, or transfer of infants and placenta to the resuscitation table(s).
- We discussed DCC (60–90 s) for the first twin, and extra-uterine placental transfusion for the second twin, but found that this would not be feasible for pilot testing. We therefore decided to exclude twins from this study.

### Protocol adjustments

Prior to pilot testing the complex intervention, the protocol was revised based on feedback from workshops and simulation sessions. Details are shown in Table 2– the INTACT-intervention.

**Table 2:** The INTACT-intervention (study protocol)

Task	Responsible
1. Prior to delivery, inform the teams about inclusion status and prepare study documents.	Midwife
2. Attach the Vacuform mattress to the mother’s legs before placing sterile drapes. Ensure enough space for SSC on the mother’s chest.	OR-circulating nurse / nurse anaesthetist
3. Prepare sterile syringes for umbilical cord blood samples, and two SteriDrape isolation bags: One for the placenta, and one for the infant (placed on a sterile towel on the mother’s legs)	Scrub nurse
4. Cover the infant in the sterile plastic bag, with the infant’s legs close to incision site. Beware of obstruction of airways or overstretching the umbilical cord.	Scrub nurse / obstetrician
5. Start Apgar timer in the moment the infant is born and announce elapsed time 10, 30 and 60 s after delivery.	OR circulating nurse
6. Attach NeoBeat on the infant’s chest within 10 s. Stimulate the infant’s back if needed. Communicate HR-readings at 10, 30 and 60 s.	Midwife
7. Record infant’s first cry/breathing status and HR-readings on the data collection sheet	OR circulating nurse
8. Obtain arterial and venous blood samples from a pulsating umbilical cord within 40 s after delivery of the infant. Communicate to time-keeper	Obstetrician
9. Deliver the placenta into the isolation bag within 60–90 s after delivery of the infant. Await signs of separation, apply gentle cord traction and uterine massage if necessary, communicate to time-keeper	Obstetrician
10. In the event of **severe maternal bleeding needing immediate attention**, communicate this to the team and consider abandoning protocol.	Obstetrician
11. Follow algorithm for newborn resuscitation: If **no spontaneous respiration**,** or HR < 100 and not rising at 30 s** after delivery, call out for early cord clamping (or early delivery of the placenta) and abandon protocol.	Midwife/ paediatric registrar
12. After delivery of the placenta, remove the outer pair of sterile gloves, ensure secure wrapping of infant and placenta, and transfer to the radiant warmer in the adjacent room (after a short welcoming cuddle if the infant is vigorous). Place the placenta next to the infant on the radiant warmer.	Midwife
13. Deliver Apgar timer and data collection sheet at the radiant warmer	OR circulating nurse
14. **For vigorous infants**: monitor heart rate by NeoBeat™ until cord clamping. Communicate HR-readings every minute, register on data collection sheet	Midwife, NICU nurse/ co-investigator
15. **For infants needing respiratory support**: replace NeoBeat with standard ECG-electrodes and pulse-oximetry for further surveillance as long as necessary.	NICU-nurse
16. Communicate HR-readings, saturation (SpO_2_) every minute). Record all readings and changes in delivered oxygen fraction (FiO_2_) on the data collection sheet	NICU-nurse/ Midwife
17. Clamp the umbilical cord when criteria for PBCC are met, at maximum 10 min after delivery. Record the time	Midwife
18. Agree on Apgar scores and duration of respiratory support before the neonatal team leave the room. Measure rectal temperature (within 10–15 min).	Midwife
19. Vigorous infant: Provide skin-to-skin contact with the mother on the operating table within 15 min after delivery. Use modified leg warmer directly the infant’s skin, add warm towels. Measure axillar temperature at 30–40 min after delivery	Midwife
20. Infant needing respiratory support after cord clamping: Transfer to the NICU. Record admission temperature.	Neonatal team
21. Estimate maternal haemorrhage by visual inspection and count of bloodstained compresses. Record result on checklist.	OR-team

OR = Operating room, NICU = Neonatal Intensive Care Unit,

### Training

We arranged twelve educational rounds for approximately 200 involved staff, covering the topics neonatal transitional physiology, DCC and ICS. Training materials included information brochures and posters, power-point presentations and templates for data collection. E-learning modules with instructional videos and standard operating procedures were available for all staff. All training activities were conducted in close collaboration with the principal investigator and co-investigator, and attendance was registered. Members of the intervention development team were responsible for training their colleagues, and 90% of the midwives, 60% of the NICU specialist nurses and 75% of the scrub nurses attended one workshop covering practical issues and data-collection tasks specific for their group. Training sessions were set up for new staff.

### Phase 2 Pilot testing the INTACT-intervention

The pilot study was conducted at Ålesund Hospital, Møre and Romsdal Hospital Trust. The hospital holds a specialised maternity unit with approximately 1200 deliveries a year. A protocol of DCC (1–3 min) was implemented in 2010. Intact-cord stabilisation using mobile resuscitation equipment became standard in vaginal births in 2018 [60], however not yet implemented in CS. The study was approved by Regional Committee for Medical Research Ethics Central Norway (REK-Midt #399101) and the institution’s internal review board.

### Participants

Women planning to give birth at Ålesund Hospital received oral and written information about the study when called for 2nd trimester ultrasound scan. They were invited to provide informed, preliminary consent for participation in the event of planned or emergency CS. Inclusion criteria were verified at admission. Women having ultrasound scans done elsewhere, and who were later referred to Ålesund hospital, were invited to participate after admission, unless delivery was imminent. Both parents were approached for informed consent on behalf of their child (exception: single parent).

Inclusion criteria: live-born singleton infants at GA 32^0^– 42^3^ weeks, delivered by CS in regional anaesthesia, informed parental consent.

Exclusion criteria: twins, significant congenital malformations, placenta complications/elevated risk of haemorrhage, complications requiring immediate CS in general anaesthesia.

After piloting for three months, involved clinicians were invited to answer a short electronic questionnaire regarding their experiences with the INTACT-intervention compared to standard care (DCC for 1–3 min, neonatal team waiting in the adjacent room). The main rationale for the survey was to investigate acceptability and inform future protocol revisions. The questionnaire was adapted from the Swedish SAVE-study [61], covering professional role, participation in training activities prior to study start, and a number of statements (Likert-scale) concerning the experience. Comments could be added in free text.

### Intervention protocol

General preparations for surgery were not altered for the study setting, with the exception of attaching a vacuum mattress to the mother’s thighs under the sterile draping. Immediately after delivery, the infant was covered in a sterile plastic bag. The attending midwife stimulated the baby and attached NeoBeat™ dry-electrode ECG over the infant’s chest. The obstetrician obtained arterial and venous samples for cord blood gas-analyses from the pulsating umbilical cord. After 60–90 s, preferably awaiting spontaneous detachment, the placenta was delivered without cutting the cord and placed in a sterile tray. Gentle cord traction and uterine massage were performed if necessary. After wrapping the infant and placenta securely, the attending midwife carried both to the resuscitation table in the adjacent room. A short welcoming cuddle for vigorous infants was allowed. Necessary stabilisation was initiated with an intact cord, following national guidelines. Umbilical cord clamping was done after the infant was breathing regularly with or without support, and the cord was white and pulseless, at maximum 10 min after delivery. The protocol would be abandoned (and ECC or early delivery of the placenta performed) to expedite transfer of the infant to the resuscitation table in the following situations:

- Severe maternal bleeding before delivery of the placenta.
- Delivery of an infant with no spontaneous respiration, or with HR < 100 and not rising at 30 s after delivery.

For more details, refer Table 2.

#### Predefined accept criteria for feasibility

Based on experience from simulation and existing hospital routines, we set the following criteria for timing of intervention components: Time from birth to placement of NeoBeat: within 10 s, blood sampling from a pulsating cord: within 40 s and time from birth to placental removal: 60–90 s. To minimize separation between mother and infant, maximum cord clamping time was set to 10 min, based on studies of duration of cord pulsations and umbilical cord blood flow in vaginal births [23, 62].

#### Predefined accept criteria for safety

Based on historical data from the electronic birth database for our institution for 2020–2022, we defined a prevalence of maximum 10% as acceptable for intraoperative maternal blood loss > 1000 ml, and prevalence of maximum 5% for infant 5-minute Apgar score < 7. WHO defines infant temperatures between 36.0 and 36.4 °C as mild hypothermia and recommend a target of 36.5–37.5 °C. Admission temperature between 36.0 and 37.5 °C for at least 90% of preterm infants admitted to NICU is used as a quality indicator by the Norwegian Neonatal Network [63]. Since we expected the majority of our cases to be term or near term, we defined a prevalence of maximum 10% as acceptable for infant rectal temperature < 36.5 °C during stabilisation.

### Data collection

Baseline characteristics, birth data and outcomes for mother and infant were extracted from the electronic birth records (CSAM NATUS v.3.5.5.15110, Oslo, Norway). Data were entered in an electronic case report form (WebCRF3), developed for the project and administered by the faculty of Medicine and Health Sciences, Norwegian University of Science and Technology. The circulating OR nurse and attending midwife (or NICU-nurse) collected data on intervention fidelity and timing of measurements, using paper-based checklists and data-collection forms. The co-investigator was present at all planned CS to assist data collection. Infant HR readings from NeoBeat™ were communicated to the team and noted on the data-collection sheet. ECG and saturation data were collected from the IntelliVue X3™ patient monitor (Phillips™) if respiratory support was given. After completing each case, the study nurses entered all data into the electronic database.

Data on clinicians’ experiences with the INTACT-intervention were collected electronically, using eFORSK, a web-based data collection system developed and administered by HEMIT (Helse Midt-Norge IT), Trondheim, Norway.

### Data analysis

#### Assessment of feasibility and acceptability

Feasibility was assessed by the proportion of successful interventions; defined as extra-uterine placental transfusion, intact cord stabilisation and PBCC successfully completed. Acceptability was assessed by summarising scores from staff questionnaires. Ratings in the categories “same”, “better” or “much better” were defined as acceptable.

#### Assessment of safety

Safety was assessed by comparing infant 5-minute Apgar scores, infant body temperatures and maternal estimated blood loss to pre-defined accept criteria. We performed an interim analysis of safety after 10 included mother-infant dyads. We closely monitored adverse events. Any concerns regarding acceptability of the different intervention components (maintenance of sterility, logistics, crowding and ergonomics) were discussed and solved by the research team (refer Lessons learned section). Sample size calculation was not done due to the observational study design with no comparison group. The advice from the statistician was to aim for a sample size of 20–30. A stop-date was set after lessons learned was summarised and decisions on further protocol revisions were made, and we ended up with a sample size of 29.

We applied IBM SPSS Statistics software (version 27) for descriptive analyses of maternal and neonatal characteristics and secondary outcomes. Spearman’s rho was estimated to analyse bivariate associations. For analyses of primary outcome and timing of intervention measures and observations, and for analyses of numerical data from the questionnaires, we used Stata Statistical Software version 18 (StataCorp). Free text comments were systematised and analysed by simple text analysis.

## Results

### Enrolment

A total of 182 women were recruited from October 3rd, 2022, to January 31st, 2023. Sixty-seven CS were conducted during this period. The first 29 mother-infant couples fulfilling inclusion criteria were included. A consort flow diagram describing the flow of participants through the study is provided as Supplementary material (Additional file 5). Characteristics of the included cases are listed in Table 3.

**Table 3:** Maternal and neonatal characteristics

Study participants (*n* = 29)	Mean/frequency	Range/percent
Mothers		
Age at CS (completed years) Higher education* Nulliparous Previous caesarean section	33 15 17 6	27–41 52% 59% 22%
Infants		
Female sex Gestational age (completed weeks + days) Birth weight (grams)	15 40 + 1 3821	52% 37 + 0–42 + 2 2800–5230
Type of regional anaesthesia		
Epidural Spinal	9 20	31% 69%
CS classification		
Planned Emergency grade 2 (within 20 min) Emergency grade 3 (within 1 h or more)	9 15 5	31% 52% 17%
Planned CS indication (*n* = 9)		
Maternal request Breech presentation Two or more previous CS Other	2 2 2 3	22% 22% 22% 34%
Emergency CS indication (*n* = 20)		
Poor progress Unsuccessful induction Suspected asphyxia Other	11 2 5 2	55% 10% 25% 10%
Observations before emergency CS (*n* = 20)		
Induction before CS Active labour before CS Rupture of membranes before CS Antibiotics before CS (if PROM > 24 h) Suspected chorionamnionitis	11 15 17 1 1	55% 75% 85%

*college or university level. CS = Caesarean Section, PROM = Pre-labour Rupture Of Membranes

### Primary outcome / feasibility

**Fig. 1:**
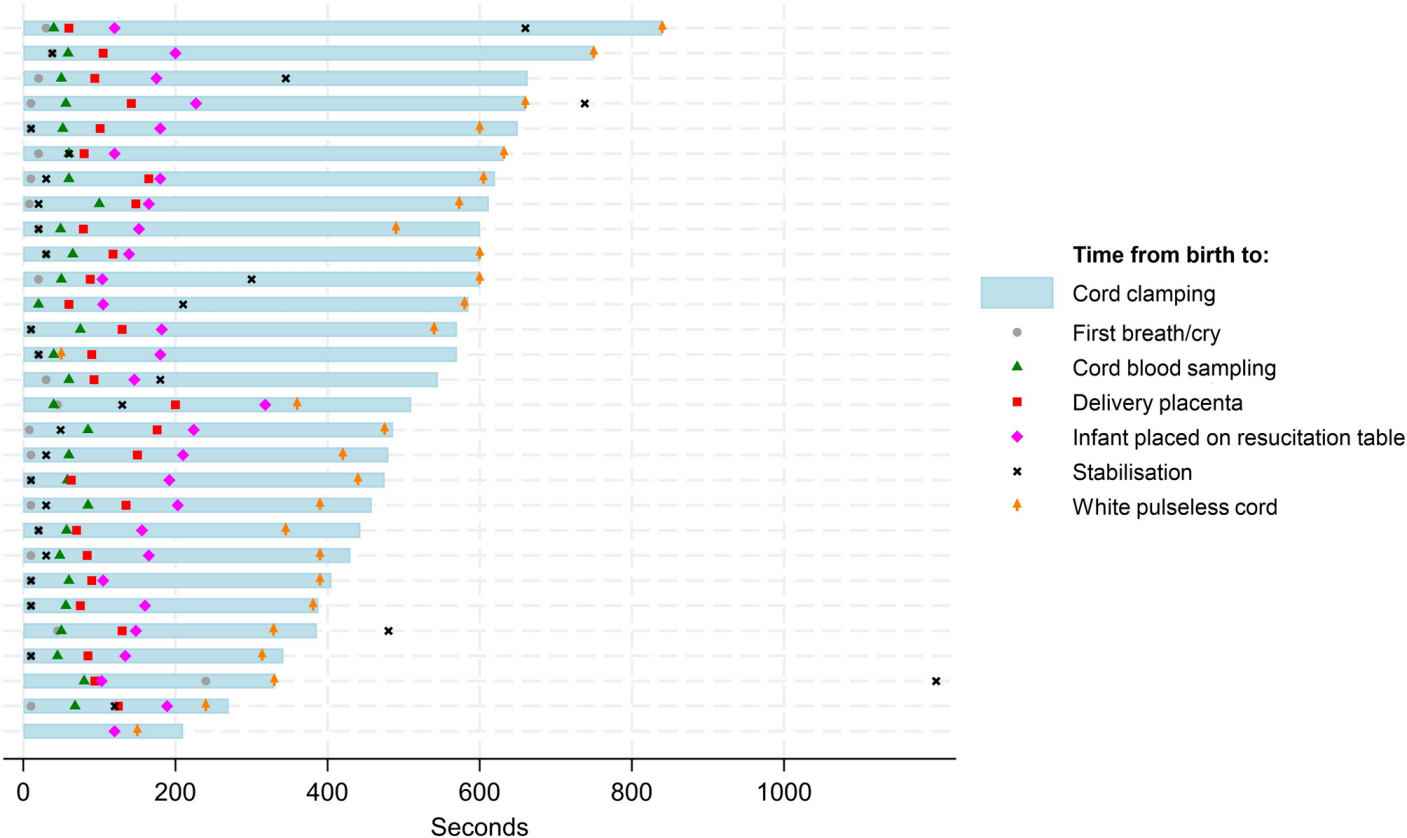
Timing of observations and measures. Study intervention components and observations registered in real time from the moment of delivery of the infant to umbilical cord clamping for all included infants in the pilot study

The combination of extra-uterine placental transfusion, intact-cord stabilisation of the infant and PBCC was completed in 26 out of 29 cases. In one infant, the cord still pulsated at cord clamping (11 min). In two cases, cord clamping was done before the infant was stabilised, although the predefined limit for cord clamping (10 min) was not reached. The mean (range) time from birth to placental delivery was 1:48 min (1:00–3:20 min). Mean cord clamping time was 8:41 min (3:30 − 14:00 min) and median time (range) from birth to stabilisation was 30 s (0:10–20:00 min). A graphical display of observations and delivery of intervention components are shown in Fig. 1.

### Acceptability

Ninety-nine of 218 eligible clinicians (45.4%) completed the survey on intervention experiences. At survey deadline (after piloting for 3 months), several clinicians had not yet participated in the pilot study. They were invited to answer survey questions based on their training experiences and how they anticipated that the INTACT-intervention would work in real life (data not shown, Additional file 4). Participants’ characteristics are shown in Table 4.

**Table 4:** Characteristics of survey participants (clinicians)

Participating clinicians	*N* = 99
Professional role	
Obstetrician/ obstetric registrar Midwife Neonatologist/Paediatrician/Paediatric registrar Neonatal intensive care nurse Anaesthesiologist Nurse anaesthetist Scrub nurse	10 25 11 20 5 4 24
Training:	
Workshop/classroom session Instruction video Simulation Other activities (supervision, demonstration) No training	82 64 12 20 4
Intervention experience	
Once More than once None	22 37 40

**Fig. 2:**
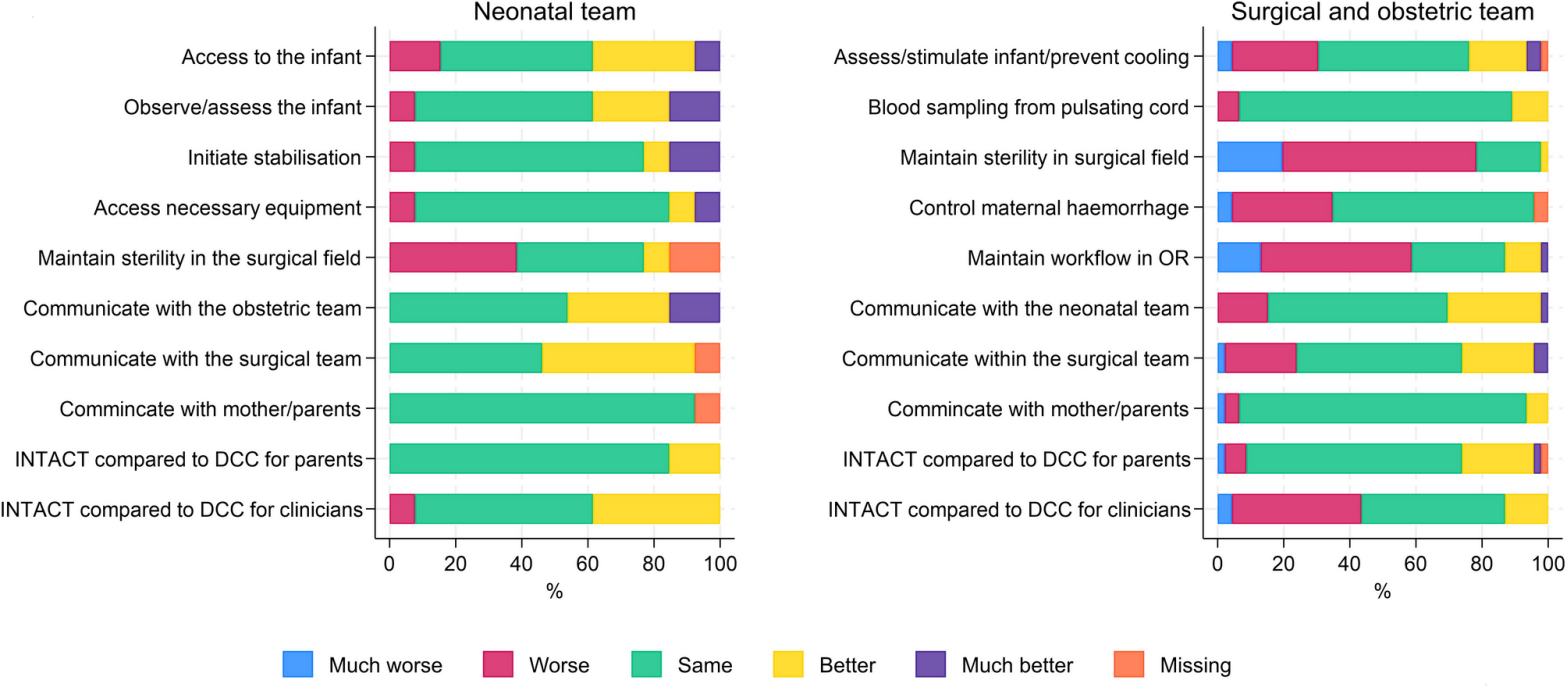
Clinicians experiences with the INTACT-intervention. Feedback on a questionnaire survey sent to all involved staff after 3 months of piloting, comparing the study intervention to standard care (DCC 1–3 min before placental delivery, neonatal team waiting in the adjacent room)

The majority (76%) of surgical and obstetric staff (*n* = 46) rated communication within the team as same, better or much better when applying the INTACT-intervention compared to standard care. For communication with the neonatal team and with the parents, the combined outcomes were 85% and 93.5% respectively. Approximately 80% of clinicians from the surgical and obstetric teams rated the ability to maintain sterility as worse or much worse with INTACT-intervention compared to standard care, mainly due to the use of non-sterile equipment (NeoBeat) in the field. The majority (75%) of neonatal staff (*n* = 31) rated the ability to observe and assess the infant as the same, better or much better with the new intervention. More details on the responses from clinicians are shown in Fig. 2.

### Secondary outcomes

Mean (range) infant temperatures were 37.0^°^C (36.4–37.8^°^C) measured rectally at 10–15 min (*n* = 23), and 36.8^°^C (36.0-37.5^°^C) measured axillary at 30–40 min for infants who had SSC with their mothers on the operating table (*n* = 16). Median (IQR) infant heartrates measured at 1, 3 and 5 min were 156 (147–184), 162 (150–174) and 167 (150–186) beats per minute (bpm) respectively. Among infants needing respiratory support (*n* = 8), one had positive pressure ventilation (PPV), the rest had mask-CPAP (Continuous Positive Airway Pressure). Median time from birth to stabilisation (HR > 100 bpm, regular breathing and saturation of ≥ 85% with delivered oxygen fraction < 40%) for this group of infants was 6:52 min (IQR 3:15 − 11:39). No infants needed full resuscitation. The median time from birth to SSC with mother on the operating table was 13 min (*n* = 19). Reasons for not attempting SSC on operating table were infant respiration support (*n* = 3), infant hypothermia (*n* = 1), maternal discomfort (*n* = 3), converting to general anaesthesia after delivery of the infant (*n* = 2), not known (*n* = 1). Other secondary outcomes are presented in Table 5.

**Table 5:** Other secondary outcomes

Infant and maternal birth data	Planned CS (*n* = 9)	Emergency CS (*n* = 20)
Apgar scores (Median, IQR)		
1 min 5 min 10 min	9 (9–10) 10 (9–10) 10 (10–10)	8 (8–9) 10 (9–10) 10 (10–10)
Umbilical cord blood gases (*n* = 22) (mean, SD)		
Arterial pH Arterial Base excess Venous pH Venous Base excess	7.33 (0.05) 1.24 (2.05) 7.34 (0.05) 1.05 (1.46)	7.27 (0.06) 2.06 (1.47) 7.33 (0.06) 2.19 (1.82)
SSC with mother (frequency, percent)		
On operating table Attempted, but abrupted During transport to recovery unit	6 (67) 1 (11) 7 (78)	10 (50) 2 (10) 7 (35)
Need for respiratory support (frequency) Transfer to NICU	1 1	7 2
Estimated maternal blood loss (ml) (mean, SD)	578 (228)	788 (445)

### Safety

Infant hypothermia (rectal temperature < 36.5 °C) during stabilisation occurred in three cases (10%). The estimated correlations between infant rectal temperature during stabilisation and timing of placental removal or timing of cord clamping were negative, but weak (rho= -0.21, *p* = 0.33 and rho = -0.11, *p* = 0.63, respectively). The correlation between infant rectal temperature during stabilisation and room temperature in OR was positive and significant (rho = 0.50, *p* = 0.03).

One infant had 5-minute Apgar score < 7, needing PPV at 4 min after birth. Low HR was observed from 30 s after birth but rising to 183 bpm at one minute. At 2 min, HR was again below 100 bpm without sign of respiration efforts. Mask-CPAP (NeoPuff) was given for 2 min without response and PPV was started. The infant recovered.

One infant receiving respiratory support did not reach the desired target range for saturation (85–95%) within 10 min, set in national guidelines for neonatal life support [58]. This infant had HR > 180 bpm for the first 12 min, and mask-CPAP was initiated at 5 min.

Three mothers (10%) had estimated intraoperative blood loss > 1000 ml, all after emergency CS. The estimated correlation between timing of placental removal and maternal blood loss was weak and not significant (rho = 0.14, *p* = 0.47). Mean haemoglobin (SD) first postoperative day (*n* = 24) was 10.3 g/dl (1.56). Three mothers (10%) received blood transfusions. There were no protocol abruptions due to excessive maternal blood loss before placental delivery.

#### Adverse events

##### Case ID 01

Severe infant hypothermia occurred during the first two hours of life despite actions to improve temperature, including feeding and warm linen. Rectal temperature fell from 36.4 to 35.6 °C.

##### Case ID 14

Bag with placenta fell to the floor during welcoming cuddle. This could potentially have caused bleeding from a ruptured cord (but did not happen).

### Lessons learned / modifications of the protocol

Survey feedback from clinicians highlighted concerns to be solved prior to further testing in a larger study:

Placement of the infant after birth:

- The sterile plastic bag (50 × 50 cm) used for prevention of infant hypothermia and contamination of the sterile field was too small for most term/near term infants.
- Difficulties covering the infant disturbed the team’s focus on bleeding from the incision site and could delay necessary simulation of the infant during transition.

Contamination of the surgical field:

- The midwife’s hands and the NeoBeat™ were not sterile and keeping them inside the sterile bag with the infant was challenging.

Disturbance of workflow for the surgical team:

- Concurrency conflicts arouse when both infant and surgical field needed attention.
- Communication about infant HR and completed tasks/observations increased noise.

Infant hypothermia

- Maintaining infant body temperature the first 2–3 h after birth was a recurring problem during the study.

We added an extra sterile drape over the mother’s legs and dropped the sterile bag for the infant. The midwife could concentrate on infant airways and HR and the extra drape could be removed to regain sterility once the infant and placenta were carried out. Based on the adverse event where a placenta fell to the floor, we increased focus on safety when transferring the infant to the resuscitation table, and informed all midwives to carry the placenta-bag in one hand or ask for assistance if both hands were needed to carry the infant. We focused on closed-loop communication and keeping conversation at a minimum to improve data collection and avoid misunderstandings. We increased focus on preventing infant hypothermia and arranged practical workshops for involved personnel. We decided to include a heating bottle for pre-heating of towels to be used for infant cover. The protocol was adjusted accordingly.

## Discussion

In this intervention development and pilot study, we were able to show that the combination of extra-uterine placental transfusion, intact cord stabilisation and PBCC was feasible in term and near-term caesarean sections performed in regional anaesthesia. However, introducing non-sterile equipment for early detection of infant HR (NeoBeat™) did occasionally compromise sterility, and disturbed workflow for the surgical team. Although maternal and infant safety outcomes were within pre-defined accept criteria, the small sample size and observational design with no control group do not allow for any definitive conclusions regarding safety.

### Timing of placental delivery

We observed difficulties with complying to predefined time frames for placental delivery, and like Welsh et al. [50], we observed variations in time from case to case. Caesarean sections have an increased risk of maternal haemorrhage, but evidence is supportive of spontaneous separation of placenta (with gentle cord traction) instead of a more active approach (manual removal) [64]. Outside the study setting, the timing is left to the preference of the obstetrician, which may have influenced the timing in our study. We defined placenta delivery as the time when placenta and membranes were separated from the uterine wall. In some cases, the membranes were difficult to remove, explaining the longer removal times. Furthermore, cord blood sampling times were also longer than the pre-specified accept criteria (within 40 s) and may have influenced the timing of placenta removal, as this was done after sampling was completed. A plausible explanation may be that obstetricians were unfamiliar with umbilical cord blood sampling, as this had formerly been done by the midwives.

### Extra-uterine placental transfusion

Although we studied the feasibility and safety of extra-uterine placental transfusion, we did not measure whether a net transfusion of blood from placenta to infant actually occurred. Several studies have demonstrated that DCC yields a net transfusion in CS, measured indirectly by increase in infant Haemoglobin or Hematocrit [34, 35, 65, 66]. One study weighed infants from birth to cord clamping for estimation of net transfused blood volume, and measured residual placental blood volume (RPBV) after DCC. Individual variations were found in transfused volume, RPBV and the duration of blood exchange resulting in a net gain [33]. We speculate that the amount of transfused blood and the duration of the blood exchange (time when infant weight stabilised) may be driven by the infants’ physiological needs, stopping when an individual equilibrium is reached. However, blood flow pattern and duration in the umbilical vessels during DCC is not fully understood.

### Intact-cord stabilisation

Although breathing problems are common after planned CS [67], and suspected asphyxia was the main indication in one of 4 emergency CS in our study, most infants in our sample were physiologically stable within 30 s. This may be explained by the maturity of the infants (37–42 weeks) and that emergency CS grade 1 (immediately, under general anaesthesia) were excluded from the study. It is possible that the INTACT-intervention may have prevented breathing problems in some of our included cases, but larger comparative studies are needed to draw conclusions on whether this approach prevents complications or benefits the infants in other aspects.

Unlike Welsh et al. [50] who investigated planned, uncomplicated CS, we included emergency CS and infants needing respiratory support in our study. We were able to show that the intervention was feasible under these circumstances. Since there were no preterm infants in the sample, we cannot draw any conclusion for this group.

### Timing of umbilical cord clamping

Our criterion for cord clamping was regular breathing AND white and pulseless cord combined, whereas other studies involving PBCC, like Brouwer et al. [68] and Knol et al. [28] have focused primarily on the infants condition (regular spontaneous breathing, heart rate > 100 bpm, SpO_2_ > 25% percentile while using FiO_2_ < 0.40). However, these studies differed from ours in that they involved very preterm infants requiring respiratory support and that the placenta was still inside the uterus at the time of cord clamping. In our study, the recorded intervals from birth to white and pulseless cord varied greatly, which corresponds with the findings in a study on term vaginal deliveries by Di Tommaso et al. [62]. However, our observations may be inaccurate since palpations for cord pulsations were done once a minute (not continuously) to minimize disturbance of blood flow. In the six cases where the cord clamping time exceeded the predefined criterion of maximum 10 min, a plausible explanation could be that cord pulsations had not yet ceased at 10 min. It could also be due to no extra personnel present to assist with cord clamping (other tasks may have been prioritised), or that the attending midwife may have considered the 10-minute limit as somewhat arbitrary.

### Early and uninterrupted skin-to-skin contact between mother and infant

In our study, median time from birth to SSC with mother on the operating Table (13 min) is longer than WHO recommendations [59, 69]. This may be attributed to the study setting (study measures and data collection to be executed before provision of SSC). Our hospital is mother-baby-friendly accredited, which means that more than 80% of infants born at our hospital shall be offered immediate and uninterrupted SSC with their mothers after birth. Compared to vaginal births, there may be other reasons for not attempting early SSC after CS, like maternal circulatory instability, dizziness and nausea, or neonatal instability requiring action. However, the frequency of SSC with mother on the operating table after CS in the study period was not different compared to the months before study start. The fraction of infants transferred to recovery unit on their mother’s chest after emergency CS increased from zero before study start to 35% during the study. Infants who did not receive SSC with their mother were offered this with the other parent as soon as possible. We recognize that keeping unnecessary separation between mother and infant at a minimum must have priority in all births, included in a study setting.

### Infant hypothermia after caesarean section

Although the prevalence of hypothermia (< 36.5 °C measured rectally) during stabilisation was within accept criteria (maximum 10%), it increased to 17% at 30–40 min. Infant hypothermia is known as a predictor of neonatal morbidity and mortality [70]. Hypothermia can be prevented by engaging in early and uninterrupted SSC between mother and infant, alternatively by ensuring that the infant is under an external heating source or covered with warm blankets [6, 70]. Nevertheless, all our cases occurred after planned CS during SSC on mother’s chest. Three of these infants had suboptimal temperatures already on the resuscitation table (under the radiant heater). Since we found a strong positive correlation between room temperatures in the OR and infant rectal temperature during stabilisation, we speculate that lower air temperatures in the OR room used for planned CS may have been a contributing factor. However, we did not adjust for possible confounders. We recognize that infants observed with hypothermia within the first 10–15 min after CS could have profited from higher room temperature and an extra heating source while in the OR, and extra attention combined with frequent temperature observations in the recovery unit.

## Conclusion

Extra-uterine placental transfusion combined with intact-cord stabilisation and physiology-based cord clamping was feasible in this pilot study of term and near-term infants delivered by CS in regional anaesthesia. We observed longer intervals from birth to placental removal, to cord clamping and to SSC than specified in the study protocol for some cases. Protocol adjustments to improve workflow and maintain sterility in the surgical field were justified, as well as measures to prevent infant hypothermia beyond stabilisation. We found no sign of harm, as prevalence of excessive intraoperative maternal blood loss, low infant 5-minute Apgar scores and hypothermia were within predefined accept criteria, but larger comparative studies, preferably including lower gestational ages are needed to establish evidence on safety of the procedure.

## Electronic supplementary material

Below is the link to the electronic supplementary material.

Supplementary Material 1

Supplementary Material 2

Supplementary Material 3

## Data Availability

The datasets used during the current study are available from the corresponding author on reasonable request.

## Abbreviations

Bpm: Beats per minute
CASP: Critical appraisal skills programme
CPAP: Continuous positive airway pressure
CS: Caesarean section
DCC: Delayed cord clamping
ECC: Early cord clamping
ECG: Electrocardiogram
ERC: European resuscitation council
FiO^2^: Delivered oxygen fraction
GA: Gestational age
HR: Heartrate
ICS: Intact-cord stabilisation
ILCOR: International Liaison Committee on Resuscitation
IQR: Inter-quartile range
MRC: Medical Research Council
NICU: Neonatal intensive care unit
OR: Operating room
PBCC: Physiology-based cord clamping
RPBV: Residual placental blood volume
SD: Standard deviation
SpO^2^: Spot oxygen concentration
SSC: Skin-to-skin contact
WHO: World Health Organization
