# Supplementary material for: Extra-uterine placental transfusion and intact-cord stabilisation of infants in caesarean sections: an intervention development and pilot-study (INTACT-1)
Source: BMC Pregnancy Childbirth. 2025 May 9;25:550. doi: 10.1186/s12884-025-07641-w (PMC12065236; doi:10.1186/s12884-025-07641-w)

**Additional file 1** PRISMA Flow diagram

Flow diagram for systematic review of the evidence on extra-uterine placental transfusion and intact-cord stabilisation during caesarean section.


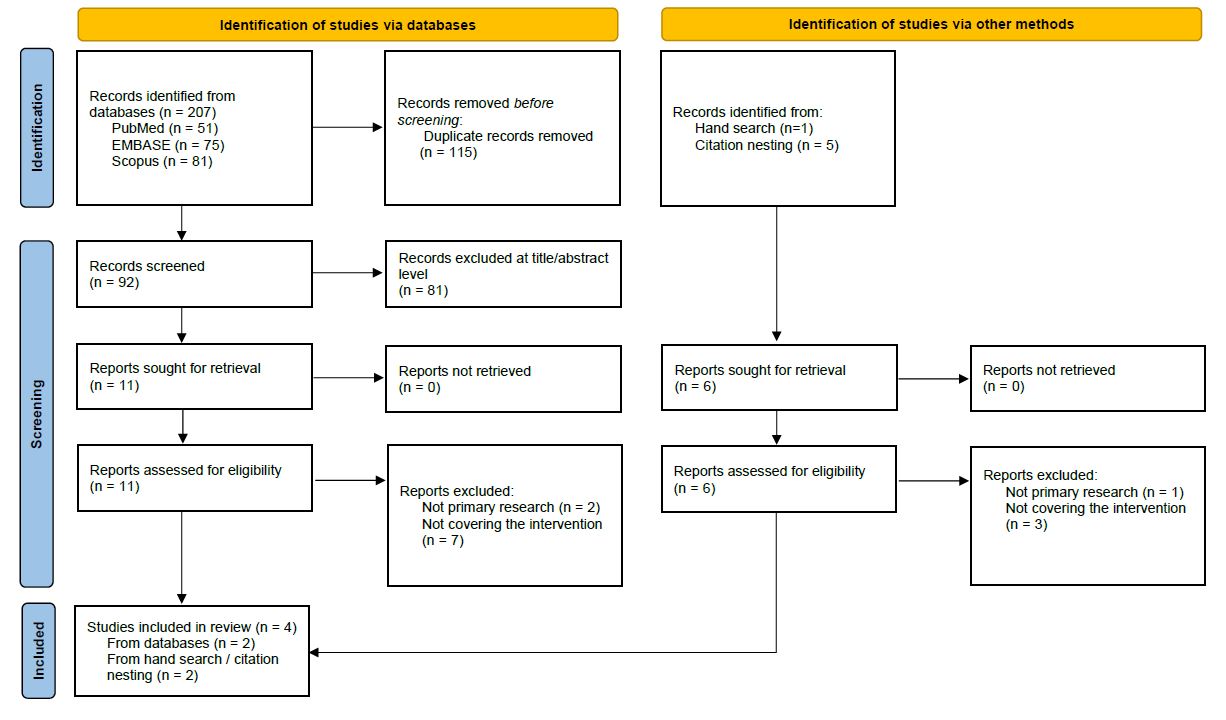


**Additional file 2** Team set-up for emergency caesarean section

Operating room overview of the team set-up for emergency caesarean section using a “pit-stop model”


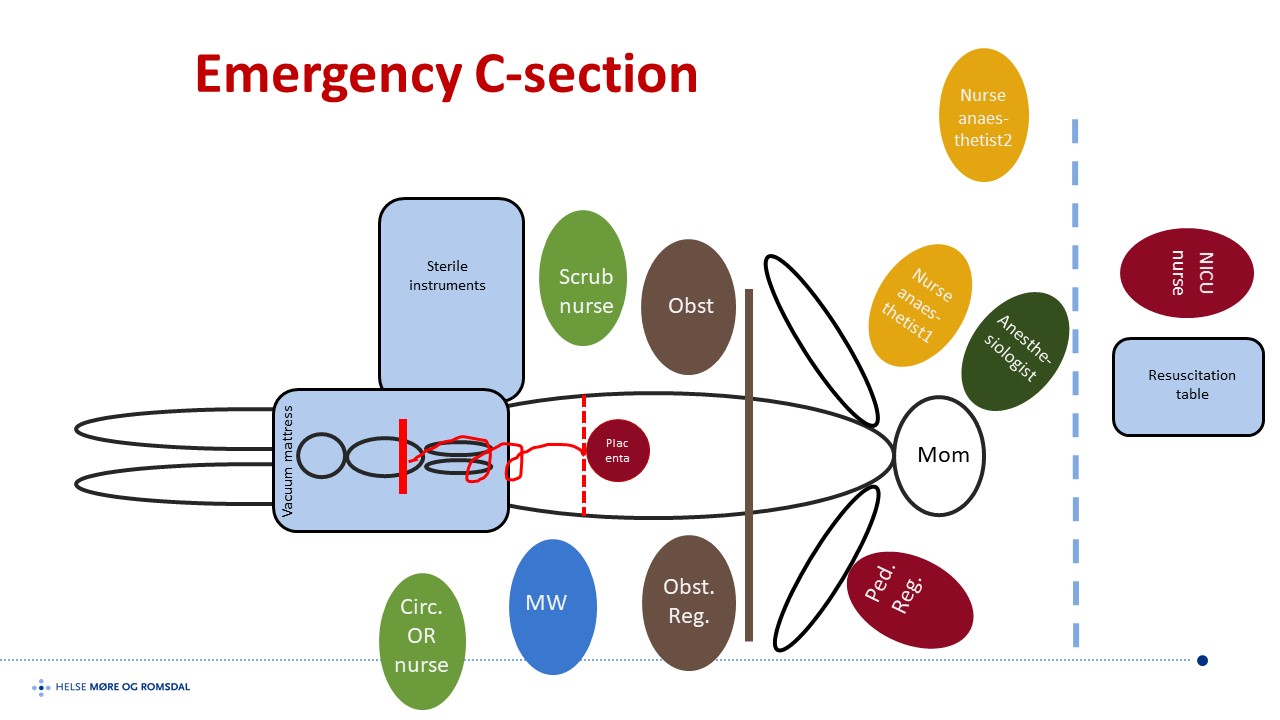


**Additional file 3** Team set-up for planned caesarean section

Operating room overview of the team set-up for planned caesarean section using a “pit-stop model”


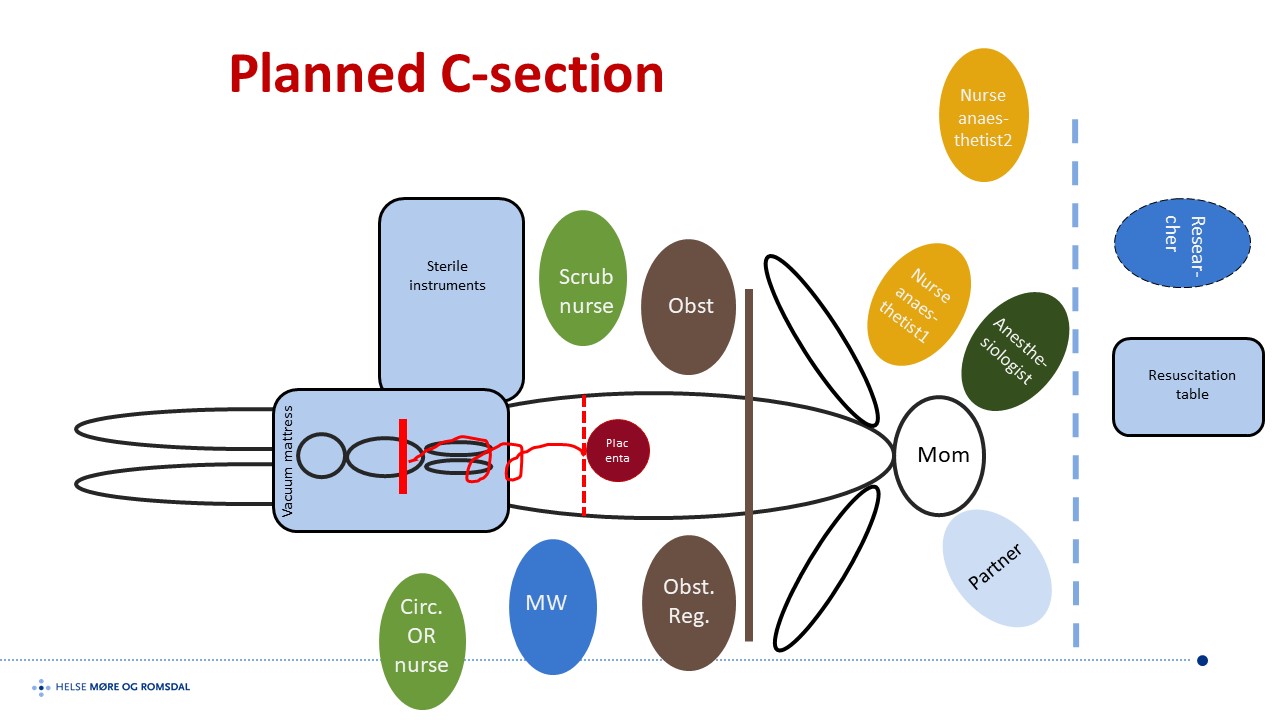


**Additional file 4** Clinicians’ experiences with the INTACT-intervention after piloting for 3 months.


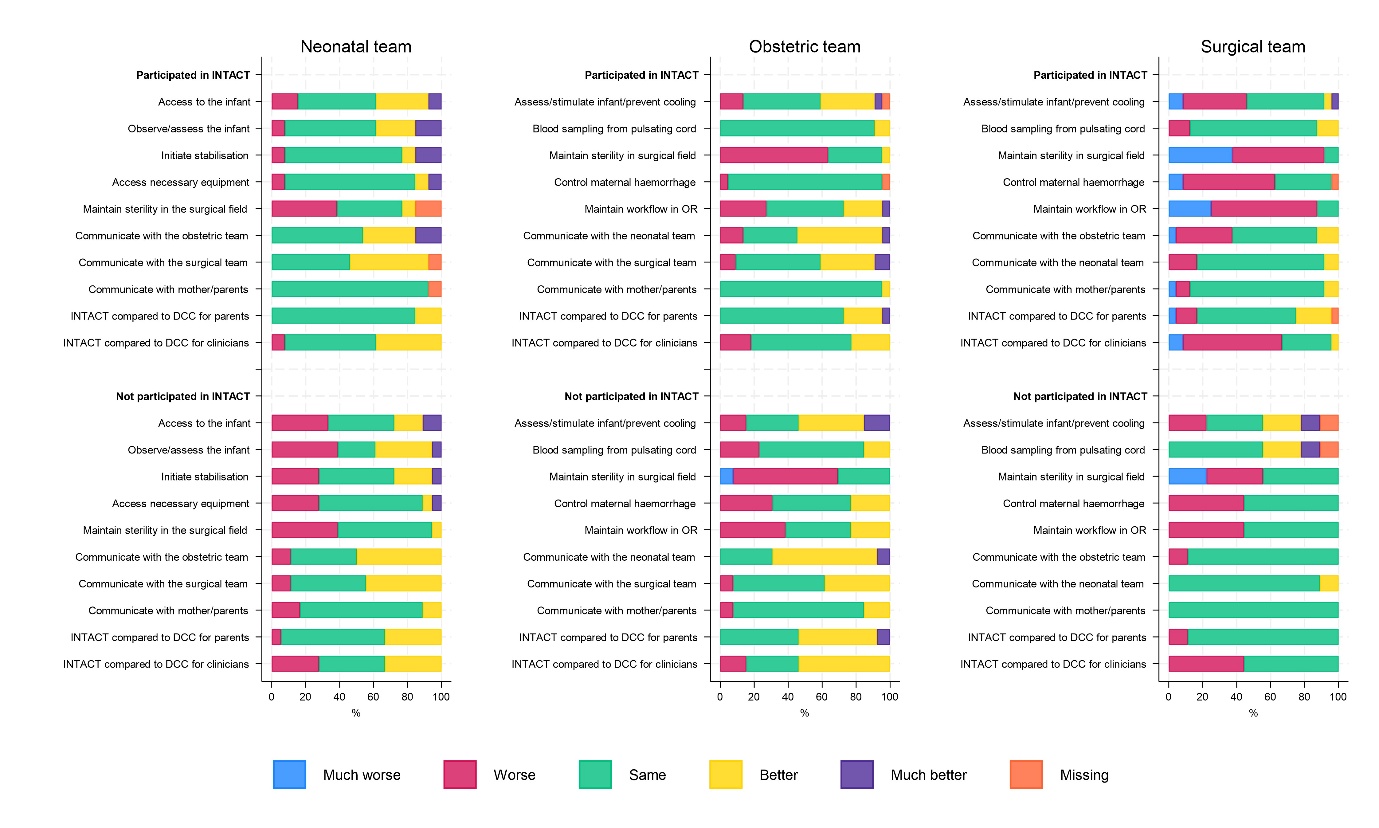


The top half shows the results from clinicians that had actually participated in the pilot-study.

The bottom half shows the results from clinicians who had not yet participated (based on their training experience)

**Additional file 5** Consort flow diagram showing the inclusion of participants
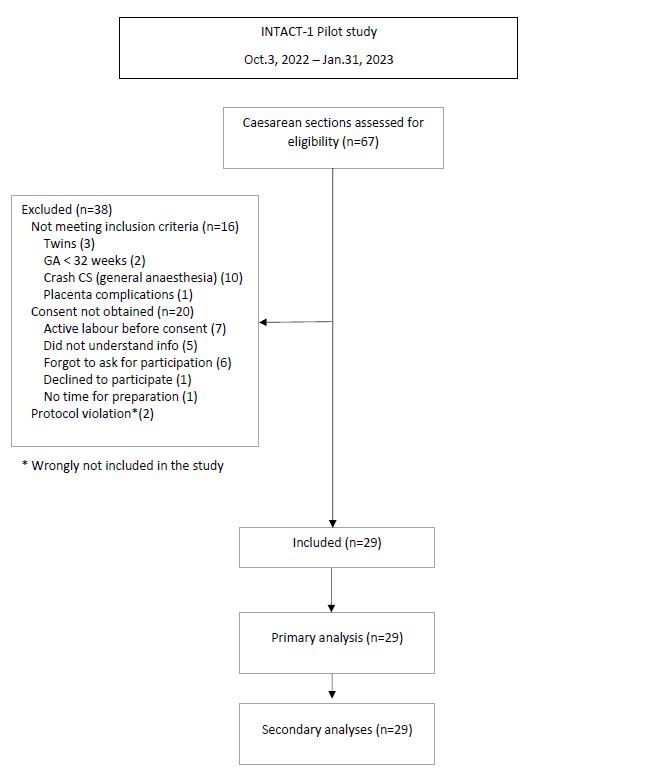

Supplement: Supplementary file 1 — Supplementary Material 1 [file 12884_2025_7641_MOESM1_ESM.docx]
